# Genomic Insights into *Listeria monocytogenes:* Organic Acid Interventions for Biofilm Prevention and Control

**DOI:** 10.3390/ijms241713108

**Published:** 2023-08-23

**Authors:** María Guadalupe Avila-Novoa, Berenice González-Torres, Jean Pierre González-Gómez, Pedro Javier Guerrero-Medina, Liliana Martínez-Chávez, Nanci Edid Martínez-Gonzáles, Cristóbal Chaidez, Melesio Gutiérrez-Lomelí

**Affiliations:** 1 Centro de Investigación en Biotecnología Microbiana y Alimentaria, Departamento de Ciencias Básicas, División de Desarrollo Biotecnológico, Centro Universitario de la Ciénega, Universidad de Guadalajara, Av. Universidad 1115, Col. Lindavista, Ocotlán 47820, Jalisco, Mexico; avila.novoa@cuci.udg.mx (M.G.A.-N.); pjgm@cuci.udg.mx (P.J.G.-M.); 2 Laboratorio Nacional para la Investigación en Inocuidad Alimentaria (LANIIA), Centro de Investigación en Alimentación y Desarrollo, A.C. (CIAD), Carretera a Eldorado Km 5.5, Campo El Diez, Culiacán 80110, Sinaloa, Mexico; berenice.gonzalez.220@estudiantes.ciad.mx (B.G.-T.); jgonzalez.219@estudiantes.ciad.mx (J.P.G.-G.); chaqui@ciad.mx (C.C.); 3 Departamentos de Farmacobiología y Matemáticas, CUCEI, Universidad de Guadalajara, Marcelino García Barragán 1451, Guadalajara 44430, Jalisco, Mexico; liliana.mchavez@academicos.udg.mx (L.M.-C.); nanci.martinez@academicos.udg.mx (N.E.M.-G.)

**Keywords:** *Listeria monocytogenes*, biofilms, resistance, virulence factors, lineage

## Abstract

*Listeria monocytogenes* is an important pathogen that has been implicated in foodborne illness. The aim of the present study was to investigate the diversity of virulence factors associated with the mechanisms of pathogenicity, persistence, and formation of biofilm *L. monocytogenes* by tandem analysis of whole-genome sequencing. The lineages that presented *L. monocytogenes* (LmAV-2, LmAV-3, and LmAV-6) from Hass avocados were lineages I and II. Listeria pathogenicity island 1 (LIPI-1) and LIPI-2 were found in the isolates, while LIPI-3 and *Listeria* genomic island (LGI-2) only was in IIb. Stress survival island (SSI-1) was identified in lineage I and II. In the in silico analysis, resistance genes belonging to several groups of antibiotics were detected, but the *bcrABC* and transposon Tn6188 related to resistance to quaternary ammonium salts (QACs) were not detected in *L. monocytogenes.* Subsequently, the anti-*L. monocytogenes* planktonic cell effect showed for QACs (MIC = 6.25 ppm/MBC = 100 ppm), lactic acid (MBC = 1 mg/mL), citric acid (MBC = 0.5 mg/mL) and gallic acid (MBC = 2 mg/mL). The anti-biofilm effect with organic acids (22 °C) caused a reduction of 4–5 log_10_ cfu/cm^2^ after 10 min against control biofilm *L. monocytogenes* formed on PP than SS. This study is an important contribution to understanding the genomic diversity and epidemiology of *L. monocytogenes* to establish a control measure to reduce the impact on the environment and the consumer.

## 1. Introduction

*Listeria monocytogenes* is an environmental pathogen that can contaminate foods and cause listerial gastroenteritis (non-invasive illness) or listeriosis (invasive illness). Listeriosis can manifest as bacteremia (if it crosses the intestinal barrier), maternal–neonatal (if it crosses the intestinal and placental barrier), and CNS (if it crosses the intestinal and blood–brain barrier) [1]. Persons with the greatest risk of listeriosis are neonates, pregnant women, adults aged 65 or older, and people who have a weakened immune system [2,3]. Additionally, the *L. monocytogenes* population structure is divided into four phylogenetic lineages (I, II, III, and IV), of which lineage I and II are responsible for many cases of listeriosis; at the same time, *L. monocytogenes* has been differentiated into 13 serotypes classified into 5 genoserotypes (or PCR groups) IIa (1/2a-3a), IIb (1/2b-3b-7), IIc (1/2c-3c), IVa (4a-4c), and IVb (4ab-4b, 4d-4e) [4,5].

In 2020, the National Outbreak Reporting System (NORS) reported 59,736 outbreaks, 2,068,586 illnesses, 43,023 hospitalizations, and 2300 deaths in the United States, where an estimated 1600 cases of listeriosis and 260 deaths each year [3,6]. The European Union reported 21 outbreaks of *L. monocytogenes* and 1 outbreak of *L. monocytogenes* serogroup IIa, 46 hospitalizations, and 12 deaths in 2021 [7].

According to the Interagency Food Safety Analytics Collaboration (IFSAC) in 2020, foodborne *L. monocytogenes* illnesses (76%) were attributed to dairy products (37.1%), fruits (24.8%), and vegetable row crops (14.1%) [8]. Some of the foods, such as fresh or frozen fruits and vegetables, dairy products, and seafood/fish, have been implicated in recalls in 2022 [9]. Moreover, ready-to-eat (RTE) foods can be considered at high risk of foodborne listeriosis, in the United States has a “zero tolerance” for RTE foods, and the criteria used for its detection and recall are ≥1 cfu in 25 g of sample [10,11].

However, the food processing environment is complex and, in particular, that of the fresh produce supply chain, associated with potential sources of *L. monocytogenes* contamination such as (i) cross-contamination from surfaces of contact food with pathogens and spoilage, (ii) improper manipulation by food handlers (iii) contamination from irrigation water, fertilization with contaminated manure and contaminated soil (agricultural practices) (iv) the processing of products (freshly cut or without heat treatment) increasing the microbiological risk [12,13]. *L. monocytogenes* has the capacity to adhere and form a biofilm to various types of food contact surfaces that are found in food processing environments, providing resistance to sanitizers and other antimicrobial agents [14]. Several studies have reported an increased antimicrobial resistance in food and environmentally recovered strains of *L. monocytogenes*. Moreover, the prevalence of antimicrobial resistance of *L. monocytogenes* is determined by antibiotic used treatment of listeriosis, high concentrations of antibiotics, the structure and composition of biofilm, limited oxygen, and nutrient accessibility, metabolic state of the cell as well as by geographical differences [15,16,17]. Moreover, persistent *L. monocytogenes* can be a source of direct and indirect contamination during to food manufacture and the environment, representing a severe concern for human health and food safety. Currently, there are developing genotyping methods with whole-genome sequencing (WGS) that allow us to know the heterogeneity of virulence in *L. monocytogenes* isolates, associated with the severity of listeriosis through multi-locus sequence-based typing, sub-categorizing distinct clonal complexes (CCs) within lineages and sublineages (SL), which is widely used for microbiological surveillance of listeriosis or continuous improvement in the implementation of control measures based on the hazard characterization. Therefore, the objectives of the present study were: (i) to determine genetic diversity and genoserotypes of *L. monocytogenes* from Hass avocados; (ii) to determine stress tolerance, presence of the resistance genes to sanitizers and antimicrobial agents and multidrug-resistant strains of *L. monocytogenes*; (iii) to evaluate the effects of treatment with organic acids for the removal of biofilm *L. monocytogenes* on polypropylene (PP) and stainless steel (SS).

## 2. Results

### 2.1. Characteristics of L. monocytogenes Sublineages and Virulence Gene

In the MLST lineage and genoserotype analysis performed on the isolates with the Institut Pasteur MLST *Listeria* database platform (Table 1), we found that the isolated LmAV-3 belongs to lineage II with genoserotype IIa, because it presents one allele for the *lmo0737* gene, also, it belongs to CC945 and sublineage SL2922. In contrast, the isolates LmAV-2 and LmAV-6 belong to lineage I with genoserotype IIb, presenting three alleles for the ORF2819 and belonging to CC3 and SL3.

**Figure 1:**
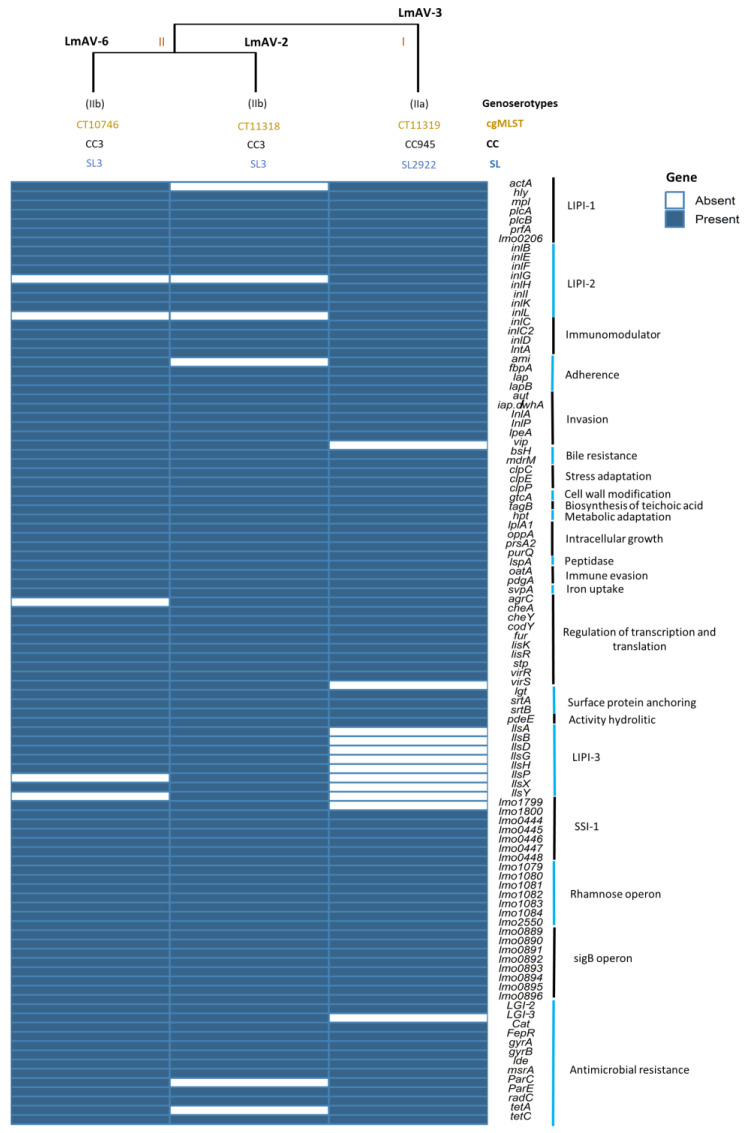
Genetic determinants of virulence of *L. monocytogenes* isolates.

In addition, in the analysis of virulence genes of *L. monocytogenes* with the virulence factor database (VFDB), we found a total of 69 genes, of which the categories that were complete in the three isolates were immunomodulator (*inlC*, *inlC2*, *inlD*, *intA*), bile resistance (*bsH*), stress adaptation (*clpCEP*), cell wall modification (*gtcA*), synthesis of teichoic acid (*tagB*), metabolic adaptation (*htp*), intracellular growth (*lplA1*, *oppA*, *prsA2*, *purQ*), peptidase (*lspA*), immune evasion (*oatA*, *pdgA*), iron uptake (*svpA*), surface protein anchoring (*lgt*, *srtAB*), activity hydrolytic (*pdeE*), peptidoglycan binding protein (*lmo1799*) and stimulate or antagonize signal transduction in a host cell (*lmo1800*), (Figure 1). Furthermore, survival and persistence are important for *L. monocytogenes*; this is achieved with the production of biofilms and their associated genes, such as *inlL*, *inlA*, *prfA*, *plcA*, *cheY*, and *prfA*, which were present in all isolates. At the same time, *actA* was absent in LmAV-2 of lineage I, and *inL* was only present in the LmAV-3 of lineage II. On the other hand, in the search for *Listeria* pathogenicity islands (LIPIs), there are absences of genes marked mainly by the type of lineage (Figure 1); for instance, the *Listeria* pathogenicity island 1 (LIPI-1) is almost complete in all isolates except for the *actA* in LmAV-2. LIPI-2 is present in all isolates but absent *inlG* and *inlL* in LmAV-2 and LmAV-6. While in the LIPI-3, it is only present in the isolates of lineage I, with absence in the genes *IIsP* and *IIsY* of LmAV-6, but complete in LmAV-2. Finally, the LIPI-4 was absent in all isolates. As for plasmids, these were found only in the isolates LmAV-2 and LmAV-6 belonging to lineage I: Inc18 (rep25) with accession number GU244485.

### 2.2. Stress Islands, Genomic Islands, Rhamnose Operon, and Silico Antimicrobial Resistance (AMR) Genes

The search of several categories was carried out on the Institut Pasteur MLST database page to understand how *L. monocytogenes* survive and persist with the ability to respond to the stress involved and the metabolic pathway for cellular composition. In Figure 1, stress survival islet SSI-1 was found in all isolates of lineages I and II, with the exception del gen *lmo1799* absent in LmAV-3 of lineage II; moreover, the isolates evaluated lack the SSI-2. Further, in the isolates of this study, LGI-1 was not found; however, some genes of LGI-2 are present in all isolates, while for LGI-3, this is absent in lineage II and present in isolates of lineage I. In addition, genes associated with rhamnose operon were present in all strains of both lineages, as well as the genes associated with the sigB operon. Finally, in the in silico analysis of genes for resistance to metals and disinfectants, the presence of resistance to compounds of quaternary ammonium salts (QACs) was not detected, such as the BC and heavy metals like cadmium (Cd) and arsenic (As). The genes involved in this resistance but that are absent in this study are BC with *bcrABC*, *emrE*, and transposon Tn6188, while for Cd is *cadAC*. Regarding the analysis of antibiotic resistance genes, the isolates showed the genes *Cat*, *Ide*, *mrsA*, *lin*, *fosX*, *mprF*, *vgaL*, *sul*, *norB*, *GyrA*, *GyrB*, *ParC*, *ParEm*, *FepR*, and *radC* with their association with resistance to chloramphenicol, ciplofloxacin, macrolide, lincosamide, ac. phosphonic, peptide, streptogramin, sulfonamides, fluoroquinolone, and efflux pumps.

### 2.3. Antimicrobial and Disinfectant Resistance Y/O Susceptibility and Reduction of Biofilm Characterization

**Figure 2:**
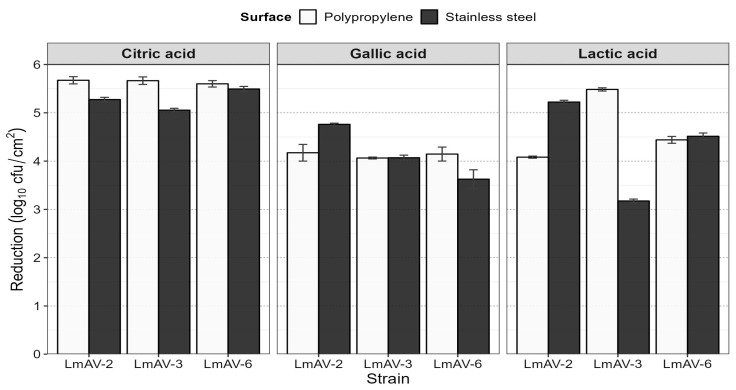
Log reductions (log_10_ cfu/cm^2^) of *L. monocytogenes* biofilm formed on polypropylene and stainless steel following 10 min disinfection at 22 °C with citric acid (2 mg/mL), gallic acid (2 mg/mL), and lactic acid (2 mg/mL).

*L. monocytogenes* strains presented multi-resistance to a wide range of groups of antibiotics such as penicillins and lincosamides (Table 2); however, LmAV-2, LmAV-3, and LmAV-6 were susceptible to ciprofloxacin, erythromycin, chloramphenicol, tetracyclines, gentamicin, trimethoprim–sulfamethoxazole, and vancomycin. Subsequently, the MBC was determined for all three strains of *L. monocytogenes* evaluated LmAV-2, and LmAV-6 was determined to be 1 mg/mL for LA; however, the MBC for LmAV-3 was 0.5 mg/mL. The MBC of all *L. monocytogenes* strains was determined to be 0.5 mg/mL for CA and 2 mg/mL GA (Table 3). The MIC of QACs was 6.25 ppm and the MBC was 100 ppm for all strains. Further, in this study, the tested microorganisms (LmAV-2, LmAV-3, and LmAV-6) showed an ability to develop biofilms in PP (8.86–8.99 log_10_ cfu/cm^2^) and SS (8.74–9.08 log_10_ cfu/cm^2^) at 240 h (*p* > 0.05). In addition, treatment with GA, CA, and LA was applied at 22 °C with one time of exposure at 10 min, determining that CA has a greater removal of biofilm of *L. monocytogenes* strains (5.46 log_10_ cfu/cm^2^; *p* < 0.05) in comparison to LA (4.48 log_10_ cfu/cm^2^) and GA (4.13 log_10_ cfu/cm^2^). Furthermore, there was no significant difference in the reduction of biofilm in LA and GA (*p* > 0.05) (Figure 2). Regarding the behavior of LmAV-2, LmAV-3, and LmAV-6 strains, there is no significant difference in the reduction of biofilm (*p* > 0.05).

## 3. Discussion

New technologies such as WGS are more frequently used since we can learn more about *L. monocytogenes*, the problem of it being present in food and causing outbreaks, the resistance it has to antibiotics and disinfectants, as well as the virulence that has and its impact on public health [18,19]. It is then that the use of these tools allows us to characterize pathogens in this study. *L. monocytogenes* strains isolated from Hass avocados are lineages I and II shown in the MLST analyses, and genoserotype IIb is the most frequent in addition to ST39 (Table 1). Other investigators have also reported similar observations of the prevalence of *L. monocytogenes* lineage I and II in various food products and food environments; moreover, these lineages have been associated with 95% human listeriosis outbreaks and sporadic cases, also suggesting that the genoserotypes harboring these lineages have a better adaptation to the human host for an increased virulence [16,20,21].

The pathogenic potential of *L. monocytogenes* is determined by the virulence factors (genes) expression to develop its pathogenicity in the host. WGS revealed that LmAV-3 (lineage II) was not detected LIPI-3 compared to LmAV-2 and LmAV-6 (lineage I) harboring most of the LIPI-1, LIPI-2 and LIPI-3 (Figure 1). LmAV-2 and LmAV-6 strains have the LIPI-3, harboring the genes *IIs* to encode Listeriolysin S (LLS), which is a bacteriocin with cytotoxic and hemolytic activity that modifies the host microbiota during infection and contributes to survival in polymorphonuclear neutrophils [15,21,22]. LIPI-3 was frequently detected in lineage I encoded LLS virulence factor implicated in severe disease [23]. A comparison in other lineages shows greater virulence in mice compared to lineage II [10,18,24]. Moreover, the isolates in this study for virulence traits showed that virulence of *L. monocytogenes* could be classified as hypovirulent since it lacks the presence of the LIPI-4 and that it encodes a transport system involved in neuronal and placental infection in the host [1].

LmAV-2 and LmAV-6 were also identified as clonal complex 3 (CC3) associated with a human clinical infection and animal listeriosis with severe manifestations. The clonal complexes (CCs) such as CC1, CC2, CC3, CC4, CC6, CC59, CC77, and CC224 of *L. monocytogenes* collected from food and clinical strains can be associated with human systemic infections and neurological forms of listeriosis [1,25]. Moreover, LmAV-3 was identified as CC945, more common in lineage II of *L. monocytogenes* strains associated with food contamination and the environment. Louha et al. [26] showed that most *L. monocytogenes* isolates from broad river watersheds belong to lineage II (68%). In these isolates, 23 different CCs were identified, with 5 CCs (CC945, CC14, CC901, CC912, and CC910) predominating. Likewise, Kim et al. [23] detected LIPI-4 in lineages I and III, and clonal complexes isolated CC4, CC87, CC213, CC217, CC363, CC382 and CC1002. Hence, the behavior of the serotypes involved in outbreaks or cases of listeriosis depends on the geographical distribution, genetic diversity, and virulence profiles of *L. monocytogenes*, epidemic clones of strains, type of food and source environments, and processing conditions in the food chain.

Other virulence factors associated with adherence, invasion, and immune evasion were detected in this study, such as the internalin family encoded by genes *inlA*, *inlB*, *inlC*, *inlF*, and *inlK* that begin with the adherence and invasion in the intestinal cells; there are other internalins such as proteins InlC, InlC2, InlD, Auto and Ami with autolysin activity [27]. In addition, some genes, like *plcA*, *iap*, and *hly*, have shown a heterogeneous presence in the analysis since this depends on the type of food matrix in which the isolates are found [28]. Temperature plays a fundamental role in the regulation and induction of the expression of virulence genes, coupled with the stress response of *L. monocytogenes* with the genes *clpCEP*, involved in both host situations (phagosome escape), as well as environmental or industrial [29]. Otherwise, the gene *hlyA*, which encodes for listeriolysin O, is responsible for the formation of the pore in the cells of the host, and this, in turn, is sought in species of *Listeria* spp. from RTE [19,30]. Also, the regulatory protein PrfA, which encodes by the gene *prfA*, is directly involved in the expression of virulence genes and acts as an activator or repressor of genes, but this depends on conditions such as high temperatures and stress [31]. In this study, *inlG* and *inlL* were not detected in lineage I. However, in lineages I and II, *inlA* encoding the inlA protein was detected in the study, which plays a role in the pathogenesis of listeriosis (invasion of human epithelial cells) or the ability of *L. monocytogenes* attachment food contact surfaces or food industry environment. In addition, premature stop codons mutation in *inlA* strains is associated with a significant attenuation of virulence or could affect biofilm formation [23,32]. Hence, the presence/absence of virulence factors (genes), is associated with the type of lineage and sequence type (ST) [10,33]. Determining the virulence potential in food is a great challenge since *L. monocytogenes* isolated from food is considered pathogenic; this virulence will depend on the serotype involved and the origin and mutation strains [34]. However, lineage II strains show higher recombination, which allows them to adapt more quickly to diverse environments, associated with several plasmids such as resistance to heavy metals, while lineage I has mechanisms that limit horizontal gene transfer [10,35]. Another way of acquiring virulence factors is through plasmids. Although it has been described that lineage II contains more plasmids than lineage I, our research shows the presence of a plasmid in both isolates LmAV-2 and LmAV-6 of lineage I, such is the case of the plasmid Inc18(rep25) pLM33, and this has been mostly associated with resistance to a wide variety of antibiotics due to their excessive use in the environment and industries and the case of plasmids belonging to lineage II, as they have a resistance to toxic components of metals and bacteriocins [10,19]. In turn, it is important to know the resistance that *L. monocytogenes* has to antimicrobials, and this can be completed phenotypically, as well as the specific search for the genes that cause it and the point mutations that exist. In the present study, the in silico analyses showed resistance to a family of antimicrobials such as lincosamide, ac. phosphonic, peptide, fluoroquinolone, streptogramin, and sulfonamides, and the genes found in all isolates with mechanisms of antibiotic inactivation (*lin*, *fosX*), target alteration (*mprF*), antibiotic efflux (*norB*), antibiotic target protection (*vgaL*) and antibiotic target replacement (*sul*). However, studies realized by Wilson et al. [36] and Mota et al. [37] mention that the gene *fosX* fosfomycin-associated resistance is present in all strains of *L. monocytogenes* due to the absence of the expression of membrane transport systems and natural resistance to lincomycin. Mafuna et al. [38] encountered resistance genes such as *fosX*, *lin*, *mprF*, and *norB*, which coincide with our results, and in turn, these have been reported as a trend in the food industry.

Regarding the in vitro analysis, *L. monocytogenes* strains were resistant to first and third generation β-lactamic (penicillin, cephalothin, ampicillin, cefotaxime, dicloxacillin) and lincosamides (clindamycin), and uniquely LmAV-3 (lineage II) are susceptible to ampicillin. Bilung et al. [39] reported the resistance to penicillin G (50–100%), ampicillin (20–100%), clindamycin (100%), and cephalothin (50–100%) in *Listeria* spp. isolated from vegetables, soil, fertilizer, and water samples collected from three farms. Likewise, Chen et al. [40] showed resistant antimicrobial to penicillin (8.1%), ampicillin (2.7%), and cephalothin (2.7%) in *L. monocytogenes* recovered from RTE foods. This trend in the resistance to β-lactamic is associated with the fact that they are antibiotics that are commonly used in the treatment of listeriosis and can be acquired or transferred antibiotic resistance genes that code for the development of mechanisms of resistance such as beta-lactamase or modification of the site to penicillin-binding protein 3 (PBP-3) in addition to the intracellular characteristic nature of *Listeria* spp. These mechanisms of resistance affect the most commonly used treatments for listeriosis, including antibiotics such as ampicillin, amoxicillin, tetracyclines, and sulfamethoxazole [41].

*L. monocytogenes* are multi-resistant for five or six antibiotics in this study but also showed susceptibility to gentamicin, erythromycin, tetracycline, vancomycin, trimethoprim–sulfamethoxazole, ciprofloxacin, and chloramphenicol which are considered as second-choice therapy in the treatment of listeriosis when there is resistance to β-lactamic or patients allergic to them [39]. Other investigators have also reported similar observations [19,20,33,42] that demonstrated the susceptibility to many antimicrobials such as sulfamethoxazole–trimethoprim, erythromycin, vancomycin, tetracycline, ciprofloxacin, chloramphenicol in *L. monocytogenes* strains recovered from humans, foods, and others.

On the other hand, there is also the challenge of the resistance generated by *L. monocytogenes* to the disinfectants that are used in the food industry and the prevention of food outbreaks that directly affect humans. In recent years, tolerance has been established towards some of the compounds used for safety, such as QACs, as well as the benzalkonium chloride (BC), which is used as an agri-food disinfectant or food environments, and resistance to it is provided by the gene cassette *bcrABC* [11,26,43]. Our study detected no genes or metal (Cd and As), *bcrABC*, and Tn6188 transposons in the three strains of *L. monocytogenes* analyzed. Moreover, all strains of *L. monocytogenes* in the study showed CMI = 6.25 ppm and CMB = 100 ppm, which gives us the guidelines for the possible efficiency as a disinfectant (BC) in the industry. In contrast, the genoserotype IIb of lineage I has *bcrABC* genes associated with resistance to BC or the genoserotype IIa of lineage II has a greater resistance to QACs, and therefore, it is present in food processing environments [26,44,45]. Muller et al. [46] attributed the resistance BC to the Tn6188 transposons. Therefore, BC is an effective disinfectant for preventing environmental niches and forming biofilm in the industry to control *L. monocytogenes* in RTE or surface food contact. However, the persistence of *L. monocytogenes* after sanitation standard operating procedure was linked to various conditions such as inadequately disinfected, difficult-to-reach locations for disinfectants, type of food contact surface, trigger stress resistance mechanism in *L. monocytogenes* by sublethal disinfectant, antimicrobial, and disinfectant resistance genes, and biofilm structure and composition [47,48]. Likewise, the presence of LGI-2 in all isolates indicated that *L. monocytogenes* has resistance to arsenic, and this coincides with that reported by Carmargo et al. [5], where that island was present in both lineages I and II, and in the case of LGI-3 with resistance to cadmium, which has recently been reported by Palma et al. [49] presenting it only isolated from ST101; however, its presence is shown in ST39 of the two lineage I strains in this study. In general, the presence of these islands has the advantage of survival and persistence of *L. monocytogenes* in complex natural or human-made environments [49,50].

All *L. monocytogenes* in this study showed the genes *inlL*, *inlA*, *prfA*, *plcA*, *agrC*, and *actA* that are involved in biofilm formation, and we found that the cell density of biofilms is 8.86–8.99 log_10_ cfu/cm^2^ in PP and 8.74–9.08 log_10_ cfu/cm^2^ in SS at 240 h (*p* > 0.05). This result suggests that strains *L. monocytogenes* can adapt to environmental conditions or adhere to food contact surfaces and form biofilm, increasing the risk of subsequent contamination of food and persistence in processing environments. Previous research indicated that genes *inlA*, *inlL*, *prfA*, *plcA*, *actA*, *Imo0673*, *bapL*, *recO*, *Imo2504*, and *luxS* play a role in biofilm formation to food contact surfaces such as stainless steel, aluminum, polycarbonate, polypropylene, polyurethane, polyvinylchloride, silicone rubber, natural white rubber, PETG, PTFE, Lexan, nitryl rubber, and glass [38,51,52]. Furthermore, biofilm is difficult to remove within the food manufacturing environment since they have important roles such as (i) facilitating mutation for developing pathogens tolerance against the antimicrobials and disinfectants, (ii) the structure and composition on the extracellular biofilm matrix confers resistance disinfectants and sanitizers, decreasing their effectiveness, which has hindered strategies for the control of biofilms within the food processing plants [47,53].

In the present study, we determined anti-*L. monocytogenes* planktonic cell effect of disinfectant showed the MBC for LA, CA, and GA (Table 3). Interestingly, the anti-biofilm effect on the CA (*p* < 0.05) has a more effective removal of biofilm of *L. monocytogenes* strains than LA and GA (Figure 2). This result agrees with several studies demonstrating that organic acids such as lactic acid, citric acid, acetic acid, ferulic acid, and gallic acid have an effect against *L. monocytogenes*, *Salmonella*, *Escherichia coli* O157:H7, *S. aureus*, and *P. aeruginosa* in food production environments [54,55]. Likewise, Borges et al. [55] argue that the GA > 5000 μg/mL had effects on the preventive action on biofilms of *L. monocytogenes* interfering with total inhibition of swimming motility of bacterium and changes in the physicochemical properties of the cell surface. The efficacy of organic acids can be associated with the increase in concentrations to be effective as decontaminating agents [56], this considering that the biofilm cells were more resistant to disinfectants than the planktonic cell [57].

This result suggests that the anti-*L. monocytogenes* planktonic cell or anti-biofilm LA, GA, and CA effect can be associated with affectation in the motility of *L. monocytogenes* which decreased the ability of the pathogen to form biofilm influencing the first step involves cells reversible attachment to surfaces and in the last stage of biofilm formation cells can detach from the biofilm and to return in planktonic cells to generate niches within food processing environmental or by affectation on the viability on the bacterium such as destabilization and permeability of cytoplasmatic membranes, bacterial type II fatty acid synthesis inhibition, efflux pump inhibition, enzyme inhibition by the oxidized [54,58,59]. All strains of *L. monocytogenes* presented the SSI-1 in this study, demonstrably previously associated with the growth and survival *L. monocytogenes* to low pH and high salt concentrations. SSI-1 and SSI-2 have been detected in isolates of *L. monocytogenes* lineages I and II, demonstrably playing a role in survival within environments such as acidic, bile, gastric, and salt stresses [5,18,49]. SSI-2 in lineages I and II are not present in the isolates in this study, so they could be less tolerant to the pH of disinfectants, making them more susceptible to organic acids and alkaline and oxidative stress. Moreover, the origin of the strains could be associated with the environment’s adaptability associated with acquired resistance mechanisms. In addition, Costa et al. [60] showed the effectiveness of acid acetic hydrogen peroxide (P3) and acid citric hydrogen peroxide (MS) disinfectants when a 4 Log reduction in viable cell persistent and nonpersistent *L. monocytogenes*, and De Jesus and Whiting [61] observed that lineage II has non-acid-adaptation, but it is more heat-resistant compared to other lineages.

Furthermore, some factors must be considered for the effectiveness of a disinfectant, such as regulatory approval, effectiveness against the pathogen, and the effectiveness of the conditions of use, such as concentration, temperature, pH, water hardness, exposure time, or application method. Organic acids such as CA, LA, and GA are within the category of generally recognized as safe (GRAS) substances. They are activity antimicrobials, considered sanitizers within the cleaning/sanitizing categories. In addition, these are not toxic to the environment and do not generate resistance [54,62] compared to various types of disinfectants used in the food industry. Moreover, to limit the persistence of niches of *L. monocytogenes* or biofilms within the industry, a disinfectant rotation plan must be implemented, of which organic acids are a proposal, in addition to the possible combination with other treatments or the inclusion of nanoparticles that enter the different states of the cells more efficiently for the control of *L. monocytogenes.*

## 4. Materials and Methods

### 4.1. Bacterial Strains

Three *L. monocytogenes* (LmAV-2, LmAV-3, and LmAV-6) strains collected from Hass avocados were selected for this study and belong to the culture collection of the Centro de Investigación en Biotecnología Microbiana y Alimentaria, Centro Universitario de la Ciénega, Universidad de Guadalajara. Stocks were stored in tryptic soy broth (TSB; Becton Dickinson Bioxon, Le Pont de Claix, France) containing 30% glycerol at −80 °C. Working cultures of *L. monocytogenes* were maintained in TSB with 0.6% yeast extract (TSBYE) (Sigma-Aldrich, St. Louis, MO, USA) for 24 h at 30 °C.

### 4.2. Genomic Characterization

#### 4.2.1. Whole-Genome Sequencing

Genomic DNA extraction from *L. monocytogenes* was performed using the kit DNeasy Blood and Tissue kit (QIAGEN, Mexico City, Mexico) according to the manufacturer’s instructions and quantified with a NanoDrop 2000c Spectrophotometer. After genomic DNA extraction, libraries were prepared with Nextera XT DNA Library Preparation Kit (Illumina, San Diego, CA, USA) according to the manufacturer’s instructions. The whole-genome sequencing for *L. monocytogenes* strains was performed with the Illumina MiniSeq platform. The genome sequences of *L. monocytogenes* were deposited in GenBank database under the accession numbers JAUNZS000000000 (LmAV-2), JAUNZR000000000 (LmAV-3), and JAUNZT000000000 (LmAV-6) in the BioProject PRJNA996147.

#### 4.2.2. Assembling and Annotation

The raw read quality was analyzed using FastQC (https://www.bioinformatics.babraham.ac.uk/projects/fastqc, accessed on 19 August 2022), and cleanup was performed with fastp v0.22.0 [63]. After filtering, the remaining reads were de novo assembled into contigs using SPAdes v3.15.3 and v3.15.4 [64]. The generated assemblies were annotated using Prokka v1.13.4.

#### 4.2.3. Bioinformatic Analyses of Whole-Genome Sequencing Data

Genome assemblies of *L. monocytogenes* strains were screened for the presence/absence of genes encoding antimicrobial resistance (AMR), sanitizers and chromosomal mutations in ResFinder v3.2 [65]. Additionally, with the ABRicate program v0.8.13 (https://github.com/tseemann/abricate, accessed on 30 August 2022), the search for resistance genes was compared with the Comprehensive Antimicrobial Resistance Database (CARD, https://card.mcmaster.ca/home, accessed on 30 August 2022). The identification of the presence/absence of virulence genes was performed using the ABRicate program v0.8.13 and compared with the virulence factor database VFDB [66]. The detection of plasmids was analyzed with PlasmidFinder 2.1 tool [67]. The Institut Pasteur MLST database (BIGSdb-Lm platform, http://bigsdb.pasteur.fr/listeria, accessed on 15 July 2022) was also used [4], and screened stress islands, genomic islands, motility, metal, and disinfectants, and rhamnose operon. The criteria were taken above 80% identity with a minimum alignment of 80%.

### 4.3. Phenotypic Characterization

#### 4.3.1. Antimicrobial Resistance and Susceptibility

The resistance and susceptibility of *L. monocytogenes* strains to antibiotics were determined using the agar diffusion method, according to the Clinical and Laboratory Standards Institute [68]. The following 13 antimicrobial agents were classified into 11 different groups according to the World Health Organization (WHO) [69]: penicillin (P: 10 U), ampicillin (AM: 10 µg), cephalothin (CF: 30 µg), cefotaxime (CFX: 30 µg), dicloxacillin (DC: 1 µg), gentamicin (GE: 10 µg), erythromycin (E: 15 µg), ciprofloxacin (CPF: 5 μg), clindamycin (CLM: 30 µg), vancomycin (VA: 30 µg), chloramphenicol (CL: 30 µg), trimethoprim–sulfamethoxazole (SXT: 2.5/23.75 μg), and tetracycline (TE: 30 µg) (BBL^TM^ Sensi-Disc^TM^). *L. monocytogenes* strains were cultured on Mueller Hinton agar (MHA; Becton Dickinson Bioxon, Le Pont de Claix, France) plates and incubated at 37 °C for 24 h. After 24 h, plates were measured in the inhibition zone, and strains were interpreted as susceptible (S), intermediate (I), or resistant (R) with reference to the standards set by the CLSI. *L. monocytogenes* ATCC 19111 was used as the positive control.

#### 4.3.2. Disinfectant Sensitivity

The citric acid (CA; Sigma-Aldrich, St. Louis, MO, USA), lactic acid (LA; Sigma-Aldrich, St. Louis, MO, USA), and gallic acid (GA; Sigma-Aldrich, St. Louis, MO, USA) were used to determine the sensitivity of *L. monocytogenes* strains (LmAV-2, LmAV-3, LmAV-6), using a broth microdilution method according to Clinical and Laboratory Standards Institute [68]. An inoculum level ~10^8^ cfu/mL was prepared for each *L. monocytogenes* strain, and different concentrations of CA, LA, and GA (4, 2, 1, 0.5, 0.25, 0.12, 0.06, 0.03, 0.01 mg/mL) were prepared in Mueller Hinton broth (MHB; Becton Dickinson Bioxon, Le Pont de Claix, France) [70]. Subsequently, 100 µL of the inoculum and 100 µL of each stock concentration of CA, LA, and GA were combined and placed in 96 well microtiter plates (Corning^®^ 96-Well Assay Microplate, Lowell, MA, USA) for a final concentration of ~10^4^ cfu/mL. All microtiter plates were incubated at 37 °C for 24. Growth was monitored for 24 h to determine the minimum inhibitory concentration (MIC) by OD_560_ using a Multiskan FC (Thermo Fisher Scientific, Inc., Madison, WI, USA). The minimum bactericidal concentration (MBC) of AC, AL, and AG were tested for any isolate with growth onto tryptic soy agar (TSA; Becton Dickinson Bioxon, Le Pont de Claix, France) and incubated at 37 °C. Alternatively, MIC and MBC were determined using BC (Sigma-Aldrich, St. Louis, MO, USA) with concentrations of 100, 50, 25, 12.5, 6.25, and 3.12 ppm [50]. Each of the assays is performed in triplicate, including positive and negative controls.

#### 4.3.3. Treatments with Organics Acids for the Reduction of Mono-Species Biofilms

Biofilms were developed on PP coupons (polypropylene type B (2 × 0.7 × 0.2 cm; Plásticos Tarkus, Guadalajara, JAL, Mexico) and stainless-steel (SS) coupons (AISI 316, 0.8 × 2.0 × 0.1 cm; CIMA Inoxidable, Guadalajara, JAL, Mexico) based on a protocol described by Avila-Novoa et al. [71]. Each coupon was individually introduced into a polypropylene tube (Corning, NY, USA) containing 5 mL of TSB. The mono-species biofilms of *L. monocytogenes* (LmAV-2, LmAV-3, and LmAV-6) were inoculated with 100 µL (~10^8^ cfu/mL) of the corresponding strain for 240 h at 37 °C. After 120 h, the coupons were removed from the tube, immersed into a new fresh medium (TSB + inoculated with the corresponding strain (~10^8^ cfu/mL)), and incubated for 120 h at 37 °C. At the end of the incubation period (240 h), the coupons were removed from polypropylene tubes under sterile conditions, and unbound cells were removed by vortexing (150 rpm for 10 s) with 5 mL of phosphate-buffered saline (PBS; Sigma-Aldrich, St. Louis, MO, USA). After the coupons with mono-species biofilms of *L. monocytogenes* were removed from the tubes using sterile forceps and immersed individually in 1.5 mL aqueous solutions of organic acids.

Mono-species biofilms of *L. monocytogenes* were treated with (i) CA in distilled H_2_O at 2 mg/mL, (ii) LA in distilled H_2_O at 2 mg/mL, and (iii) GA in distilled H_2_O and glycerol (Sigma-Aldrich, St. Louis, MO, USA) at 2 mg/mL. The CA, LA, and GA were applied at 22 °C with one exposure time at 10 min. After the exposure period, each coupon was transferred to 1.5 mL of Dey/Engley broth (D/E; Becton Dickinson Bioxon, Le Pont de Claix, France), the neutralizing agent for disinfectants, for 30 min. Bacterial enumeration was estimated by standard plate counting on TSA (Becton Dickinson Bioxon, Le Pont de Claix, France) at 37 °C/24 h and epifluorescence microscopy of biofilms was completed after incubation was conducted as previously described by Avila-Novoa et al. [71]. Each assay was performed in triplicate, and controls with distilled water and glycerol were included.

### 4.4. Statistical Analysis

All experiments were evaluated using analysis of variance (ANOVA), followed by a least significant difference (LDS) test, in the Statgraphics Centurion XVI software program (StatPoint Technologies, Inc., The Plains, VA, USA). Values of *p* < 0.05 were considered statistically significant.

## 5. Conclusions

The results contribute to determining the pathogenic potential of the genomic diversity of *L. monocytogenes* from a food safety and public health perspective. Identifying the virulence factors related to the pathogenicity and severity of its pathology, genes involved in the survival and persistence, as well as the detection absence/presence of antimicrobials and disinfectants resistance-encoding through the use of tools such as WGS, allow us to perform detection traceability and be able to know the evolutionary changes in *L. monocytogenes*. Furthermore, this study reinforces the potential of organic acids as control measures within the industry, which could have less impact on the environment and decreased resistance to sanitizing. Additionally, future research should consider *L. monocytogenes* focus on (i) the persistence of *L. monocytogenes* in the RTE processing environment (food contact surfaces, water, and soil), (ii) transfer and survival of the pathogen in fresh products considering plant conditions and product marketing, and (iii) the effect of organic residues and nanoparticles on the effectiveness of a disinfectant.

## Figures and Tables

**Table 1:** Identification of *L. monocytogenes* lineage.

Strain	Species	Lineage	Sublineage	Genoserotypes	Clonal Complex	ST
LmAV-3	*L. monocytogenes*	II	SL2922	IIa	CC945	2922
LmAV-2	*L. monocytogenes*	I	SL3	IIb	CC3	39
LmAV-6	*L. monocytogenes*	I	SL3	IIb	CC3	39

ST, Sequence types.

**Table 2:** Results of antimicrobial susceptibility test of *L. monocytogenes* according to antimicrobial group.

Antimicrobial Group	Antibiotic	Antimicrobial Class According to the WHO ^a^	No. (%) of *L. monocytogenes*	
			Susceptible	Resistant
Penicillins	Penicillin	CI	33.3	100
	Ampicillin	CI		66.6
	Dicloxacillin	CI		100
Aminoglycosides	Gentamicin	CI	100	
Macrolides	Erythromycin	CI	100	
Quinolones	Ciprofloxacin	CI	100	
Glycopeptides	Vancomycin	CI	100	
Cephalosporines (3rd generation)	Cefotaxime	CI		100
Amphenicols	Chloramphenicol	HI	100	
Lincosamides	Clindamycin	HI		100
Tetracyclines	Tetracycline	HI	100	
Sulfonamides, dihydrofolate reductase inhibitors and combinations	Trimethoprim– sulfamethoxazole	HI	100	
Cephalosporines (1st generation)	Cephalothin	HI		100

^a^ CI, critically important; Hl, highly important.

**Table 3:** Characteristics of antimicrobial and disinfectant resistance profile of *L. monocytogenes*.

Strains	Resistance	Susceptible	LA (mg/mL)	CA (mg/mL)	GA (mg/mL)
LmAV-3	PE, CF, CFX, DC, CLM	AM, CPF, E, GE, TE, STX, VA, CL	MIC = 0.12	MIC = 0.12	MIC = 0.25
			MBC = 0.50	MBC = 0.50	MBC = 2.00
LmAV-2	PE, CF, AM, CFX, DC, CLM	CPF, E, GE, TE, STX, VA, CL	MIC = 0.12	MIC = 0.12	MIC = 0.25
			MBC = 1.00	MBC = 0.50	MBC = 2.00
LmAV-6	PE, CF, AM, CFX, DC, CLM	CPF, E, GE, TE, STX, VA, CL	MIC = 0.12	MIC = 0.12	MIC = 0.25
			MBC = 1.00	MBC = 0.50	MBC = 2.00

AM, ampicillin; CLM, clindamycin; CF, cephalothin; CFX, cefotaxime; CPF, ciprofloxacin; CL, chloramphenicol; GE, gentamicin; E, erythromycin; TE, tetracycline; VA, vancomycin; SXT, trimethoprim–sulfamethoxazole; PE, penicillin; DC, dicloxacillin; MIC, minimum inhibitory concentration; MBC, minimum bactericidal concentration; CA, citric acid; LA, lactic acid; GA, gallic acid.

## Data Availability

The genome sequences of *L. monocytogenes* were deposited in GenBank database under the accession numbers JAUNZS000000000 (LmAV-2), JAUNZR000000000 (LmAV-3), and JAUNZT000000000 (LmAV-6) in the BioProject PRJNA996147.

## References

[1] Maury M.M., Tsai Y.H., Charlier C., Touchon M., Chenal-Francisque V., Leclercq A., Criscuolo A., Gaultier C., Roussel S., Brisabois A. (2016). Uncovering *Listeria monocytogenes* hypervirulence by harnessing its biodiversity. Nat. Genet..

[2] Radoshevich L., Cossart P. (2018). *Listeria monocytogenes*: Towards a complete picture of its physiology and pathogenesis. Nat. Rev. Microbiol..

[3] Centers for Disease Control and Prevention (CDC) *Listeria* (Listeriosis). https://www.cdc.gov/listeria/index.html.

[4] Moura A., Criscuolo A., Pouseele H., Maury M.M., Leclercq A., Tarr C., Bjorkman J.T., Dallman T., Reimer A., Enouf V. (2016). Whole genome-based population biology and epidemiological surveillance of *Listeria monocytogenes*. Nat. Microbiol..

[5] Camargo A.C., Moura A., Avillan J., Herman N., McFarland A.P., Sreevatsan S., Call D.R., Woodward J.J., Lecuit M., Nero L.A. (2019). Whole-genome sequencing reveals *Listeria monocytogenes* diversity and allows identification of long-term persistent strains in Brazil. Environ. Microbiol..

[6] National Outbreak Reporting System (NORS). https://wwwn.cdc.gov/norsdashboard/.

[7] European Food Safety Authority (EFSA) Foodborne Outbreaks—Dashboard. https://www.efsa.europa.eu/en/microstrategy/FBO-dashboard.

[8] Interagency Food Safety Analytics Collaboration (IFSAC) Foodborne Illness Source Attribution Estimates for 2020 for *Salmonella*, *Escherichia coli* O157, and *Listeria monocytogenes* Using Multi-Year Outbreaks Surveillance Data, United States. https://www.cdc.gov/foodsafety/ifsac/pdf/P19-2020-report-TriAgency-508.pdf.

[9] Food & Drug (FDA) Recalls, Market Withdrawals, & Safety Alerts. https://www.fda.gov/safety/recalls-market-withdrawals-safety-alerts.

[10] Orsi R.H., den Bakker H.C., Wiedmann M. (2011). *Listeria monocytogenes* lineages: Genomics, evolution, ecology, and phenotypic characteristics. Int. J. Med. Microbiol..

[11] Food & Drug (FDA) Control of *Listeria monocytogenes* in Ready-to-Eat Food: Guidande for Industry Draff Guidante. https://www.fda.gov/media/102633/download.

[12] Smith A., Hearn J., Taylor C., Wheelhouse N., Kaczmarek M., Moorhouse E., Singleton I. (2019). *Listeria monocytogenes* isolates from ready to eat plant produce are diverse and have virulence potential. Int. J. Food Microbiol..

[13] García-Frutos R., Martínez-Chávez L., Cabrera-Díaz E., Gutiérrez-González P., Montañez-Soto J.L., Varela-Hernández J.J., Martínez-Gonzáles N.E. (2020). *Salmonella*, *Listeria monocytogenes*, and indicator microorganisms on hass avocados sold at retail markets in Guadalajara, Mexico. J. Food Prot..

[14] Abeysundara P.D.A., Dhowlaghar N., Nannapaneni R., Schilling M.W., Chang S., Mahmoud B., Sharma C.S., Ma D.P. (2017). Growth and biofilm formation by *Listeria monocytogenes* in cantaloupe flesh and peel extracts on four food-contact surfaces at 22 °C and 10 °C. Food Control.

[15] Cotter P.D., Draper L.A., Lawton E.M., Daly K.M., Groeger D.S., Casey P.G., Ross R.P., Hill C. (2008). Listeriolysin S, a novel peptide hemolysin associated with a subset of lineage I *Listeria monocytogenes*. PLoS Pathog..

[16] Wieczorek K., Osek J. (2017). Prevalence, genetic diversity and antimicrobial resistance of *Listeria monocytogenes* isolated from fresh and smoked fish in Poland. Food Microbiol..

[17] Asma S.T., Imre K., Morar A., Herman V., Acaroz U., Mukhtar H., Arslan-Acaroz D., Shah S.R.A., Gerlach R. (2022). An Overview of Biofilm Formation–Combating Strategies and Mechanisms of Action of Antibiofilm Agents. Life.

[18] Hurley D., Luque-Sastre L., Parker C.T., Huynh S., Eshwar A.K., Nguyen S.V., Andrews N., Moura A., Fox E.M., Jordan K. (2019). Whole-Genome Sequencing-Based Characterization of 100 *Listeria monocytogenes* Isolates Collected from Food Processing Environments over a Four-Year Period. mSphere.

[19] Parra-Flores J., Holy O., Bustamante F., Lepuschitz S., Pietzka A., Contreras-Fernandez A., Castillo C., Ovalle C., Alarcon-Lavin M.P., Cruz-Cordova A. (2021). Virulence and Antibiotic Resistance Genes in *Listeria monocytogenes* Strains Isolated From Ready-to-Eat Foods in Chile. Front. Microbiol..

[20] Sosnowski M., Lachtara B., Wieczorek K., Osek J. (2019). Antimicrobial resistance and genotypic characteristics of *Listeria monocytogenes* isolated from food in Poland. Int. J. Food Microbiol..

[21] Chen Y., Chen M., Wang J., Wu Q., Cheng J., Zhang J., Sun Q., Xue L., Zeng H., Lei T. (2020). Heterogeneity, Characteristics, and Public Health Implications of *Listeria monocytogenes* in Ready-to-Eat Foods and Pasteurized Milk in China. Front. Microbiol..

[22] Quereda J.J., Meza-Torres J., Cossart P., Pizarro-Cerdá J. (2017). Listeriolysin S: A bacteriocin from epidemic *Listeria monocytogenes* strains that targets the gut microbiota. Gut Microbes.

[23] Kim S.W., Haendiges J., Keller E.N., Myers R., Kim A., Lombard J.E., Karns J.S., Van Kessel J.A.S., Haley B.J. (2018). Genetic diversity and virulence profiles of *Listeria monocytogenes* recovered from bulk tank milk, milk filters, and milking equipment from dairies in the United States (2002 to 2014). PLoS ONE.

[24] Vines A., Swaminathan B. (1998). Identification and characterization of nucleotide sequence differences in three virulence-associated genes of *Listeria monocytogenes* strains representing clinically important serotypes. Curr. Microbiol..

[25] Yin Y., Yao H., Doijad S., Kong S., Shen Y., Cai X., Tan W., Wang Y., Feng Y., Ling Z. (2019). A hybrid sub-lineage of *Listeria monocytogenes* comprising hypervirulent isolates. Nat. Commun..

[26] Louha S., Meinersmann R.J., Abdo Z., Berrang M.E., Glenn T.C. (2020). An Open-Source Program (Haplo-ST) for Whole-Genome Sequence Typing Shows Extensive Diversity among *Listeria monocytogenes* Isolates in Outdoor Environments and Poultry Processing Plants. Appl. Environ. Microbiol..

[27] Hamon M., Bierne H., Cossart P. (2006). *Listeria monocytogenes*: A multifaceted model. Nat. Rev. Microbiol..

[28] Rantsiou K., Mataragas M., Alessandria V., Cocolin L. (2012). Expression of Virulence Genes of *Listeria monocytogenes* in Food. J. Food Saf..

[29] Leimeister-Wachter M., Domann E., Chakraborty T. (1992). The expression of virulence genes in *Listeria monocytogenes* is thermoregulated. J. Bacteriol..

[30] Churchill R.L., Lee H., Hall J.C. (2006). Detection of *Listeria monocytogenes* and the toxin listeriolysin O in food. J. Microbiol. Methods.

[31] Gaballa A., Guariglia-Oropeza V., Wiedmann M., Boor K.J. (2019). Cross Talk between *SigB* and *PrfA* in *Listeria monocytogenes* Facilitates Transitions between Extra- and Intracellular Environments. Microbiol. Mol. Biol. Rev..

[32] Buchanan R.L., Gorris L.G.M., Hayman M.M., Jackson T.C., Whiting R.C. (2017). A review of *Listeria monocytogenes*: An update on outbreaks, virulence, dose-response, ecology, and risk assessments. Food Control.

[33] Zhang Y., Dong S., Chen H., Chen J., Zhang J., Zhang Z., Yang Y., Xu Z., Zhan L., Mei L. (2019). Prevalence, Genotypic Characteristics and Antibiotic Resistance of *Listeria monocytogenes* From Retail Foods in Bulk in Zhejiang Province, China. Front. Microbiol..

[34] Severino P., Dussurget O., Vêncio R.Z., Dumas E., Garrido P., Padilla G., Piveteau P., Lemaître J.P., Kunst F., Glaser P. (2007). Comparative transcriptome analysis of *Listeria monocytogenes* strains of the two major lineages reveals differences in virulence, cell wall, and stress response. Appl. Environ. Microbiol..

[35] Pirone-Davies C., Chen Y., Pightling A., Ryan G., Wang Y., Yao K., Hoffmann M., Allard M.W. (2018). Genes significantly associated with lineage II food isolates of *Listeria monocytogenes*. BMC Genom..

[36] Wilson A., Gray J., Chandry P.S., Fox E.M. (2018). Phenotypic and Genotypic Analysis of Antimicrobial Resistance among *Listeria monocytogenes* Isolated from Australian Food Production Chains. Genes.

[37] Mota M.I., Vazquez S., Cornejo C., D’Alessandro B., Braga V., Caetano A., Betancor L., Varela G. (2020). Does Shiga Toxin-Producing *Escherichia coli* and *Listeria monocytogenes* Contribute Significantly to the Burden of Antimicrobial Resistance in Uruguay?. Front. Vet. Sci..

[38] Mafuna T., Matle I., Magwedere K., Pierneef R.E., Reva O.N. (2021). Whole Genome-Based Characterization of *Listeria monocytogenes* Isolates Recovered From the Food Chain in South Africa. Front. Microbiol..

[39] Bilung L.M., Chai L.S., Tahar A.S., Ted C.K., Apun K. (2018). Prevalence, Genetic Heterogeneity, and Antibiotic Resistance Profile of *Listeria* spp. and *Listeria monocytogenes* at Farm Level: A Highlight of ERIC- and BOX-PCR to Reveal Genetic Diversity. Biomed. Res. Int..

[40] Chen M., Wu Q., Zhang J., Yan Z., Wang J. (2014). Prevalence and characterization of *Listeria monocytogenes* isolated from retail-level ready-to-eat foods in South China. Food Control.

[41] Prieto M., Martínez C., Aguerre L., Rocca M.F., Cipolla L., Callejo R. (2016). Antibiotic susceptibility of *Listeria monocytogenes* in Argentina. Enferm. Infecc. Microbiol. Clin..

[42] Boháčová M., Zdeňková K., Tomáštíková Z., Fuchsová V., Demnerová K., Karpíšková R., Pazlarová J. (2018). Monitoring of resistance genes in *Listeria monocytogenes* isolates and their presence in the extracellular DNA of biofilms: A case study from the Czech Republic. Folia Microbiol..

[43] López-Alonso V., Ortiz S., Corujo A., Martínez-Suárez J.V. (2020). Analysis of Benzalkonium chloride resistance and potential virulence of *Listeria monocytogenes* isolates obtained from different stages of a poultry production chain in Spain. J. Food Prot..

[44] Poimenidou S.V., Chrysadakou M., Tzakoniati A., Bikouli V.C., Nychas G.J., Skandamis P.N. (2016). Variability of *Listeria monocytogenes* strains in biofilm formation on stainless steel and polystyrene materials and resistance to peracetic acid and quaternary ammonium compounds. Int. J. Food Microbiol..

[45] Cooper A.L., Carrillo C.D., DeschEnes M., Blais B.W. (2021). Genomic Markers for Quaternary Ammonium Compound Resistance as a Persistence Indicator for *Listeria monocytogenes* Contamination in Food Manufacturing Environments. J. Food Prot..

[46] Muller A., Rychli K., Muhterem-Uyar M., Zaiser A., Stessl B., Guinane C.M., Cotter P.D., Wagner M., Schmitz-Esser S. (2013). Tn*6188*—A novel transposon in *Listeria monocytogenes* responsible for tolerance to benzalkonium chloride. PLoS ONE.

[47] Bogino P.C., de las Mercedes-Oliva M., Sorroche F.G., Giordano W. (2013). The role of bacterial biofilms and surface components in plant-bacterial associations. Int. J. Mol. Sci..

[48] Bansal M., Dhowlaghar N., Nannapaneni R., Kode D., Chang S., Sharma C.S., McDaniel C., Kiess A. (2021). Decreased biofilm formation by planktonic cells of *Listeria monocytogenes* in the presence of sodium hypochlorite. Food Microbiol..

[49] Palma F., Brauge T., Radomski N., Mallet L., Felten A., Mistou M.Y., Brisabois A., Guillier L., Midelet-Bourdin G. (2020). Dynamics of mobile genetic elements of *Listeria monocytogenes* persisting in ready-to-eat seafood processing plants in France. BMC Genom..

[50] Gray J.A., Chandry P.S., Kaur M., Kocharunchitt C., Bowman J.P., Fox E.M. (2021). Characterisation of *Listeria monocytogenes* food-associated isolates to assess environmental fitness and virulence potential. Int. J. Food Microbiol..

[51] Kumar S., Parvathi A., George J., Krohne G., Karunasagar I., Karunasagar I. (2009). A study on the effects of some laboratory-derived genetic mutations on biofilm formation by *Listeria monocytogenes*. World J. Microbiol. Biotechnol..

[52] Travier L., Guadagnini S., Gouin E., Dufour A., Chenal-Francisque V., Cossart P., Olivo-Marin J.C., Ghigo J.M., Disson O., Lecuit M. (2013). ActA Promotes *Listeria monocytogenes* Aggregation, Intestinal Colonization and Carriage. PLoS Pathog..

[53] Hall C.W., Mah T.F. (2017). Molecular mechanisms of biofilm-based antibiotic resistance and tolerance in pathogenic bacteria. FEMS Microbiol. Rev..

[54] Borges A., Saavedra M.J., Simões M. (2012). The activity of ferulic and gallic acids in biofilm prevention and control of pathogenic bacteria. Biofouling.

[55] Valiolahi M., Najafi M.A., Eskandani M.A., Rahnama M. (2019). Effects of organic acid alone and in combination with H_2_O_2_ and NaCl on *Escherichia coli* O157:H7: An evaluation of antioxidant retention and overall acceptability in Basil leaves (*Ocimum basilicum*). Int. J. Food Microbiol..

[56] Siragusa G.R. (1995). The Effectiveness of Carcass Decontamination Systems for Controlling the Presence of Pathogens on the Surfaces of Meat Animal Carcasses. J. Food Saf..

[57] Chaitiemwong N., Hazeleger W.C., Beumer R.R. (2014). Inactivation of *Listeria monocytogenes* by disinfectants and bacteriophages in suspension and stainless steel carrier tests. J. Food Prot..

[58] Campos F.M., Couto J.A., Figueiredo A.R., Tóth I.V., Rangel A.O.S.S., Hogg T.A. (2009). Cell membrane damage induced by phenolic acids on wine lactic acid bacteria. Int. J. Food Microbiol..

[59] Simões M., Bennett R.N., Rosa E.A. (2009). Understanding antimicrobial activities of phytochemicals against multidrug resistant bacteria and biofilms. Nat. Prod. Rep..

[60] Costa A., Bertolotti L., Brito L., Civera T. (2016). Biofilm Formation and Disinfectant Susceptibility of Persistent and Nonpersistent *Listeria monocytogenes* Isolates from Gorgonzola Cheese Processing Plants. Foodborne Pathog. Dis..

[61] De Jesus A.J., Whiting R.C. (2003). Thermal inactivation, growth, and survival studies of *Listeria monocytogenes* strains belonging to three distinct genotypic lineages. J. Food Prot..

[62] Shao D., Li J., Li J., Tang R., Liu L., Shi J., Huang Q., Yang H. (2015). Inhibition of Gallic Acid on the Growth and Biofilm Formation of *Escherichia coli* and *Streptococcus mutans*. J. Food Sci..

[63] Chen S., Zhou Y., Chen Y., Gu J. (2018). fastp: An ultra-fast all-in-one FASTQ preprocessor. Bioinformatics.

[64] Bankevich A., Nurk S., Antipov D., Gurevich A.A., Dvorkin M., Kulikov A.S., Lesin V.M., Nikolenko S.I., Pham S., Prjibelski A.D. (2012). SPAdes: A new genome assembly algorithm and its applications to single-cell sequencing. J. Comput. Biol..

[65] Zankari E., Hasman H., Cosentino S., Vestergaard M., Rasmussen S., Lund O., Aarestrup F.M., Larsen M.V. (2012). Identification of acquired antimicrobial resistance genes. J. Antimicrob. Chemother..

[66] Liu B., Zheng D., Jin Q., Chen L., Yang J. (2019). VFDB 2019: A comparative pathogenomic platform with an interactive web interface. Nucleic Acids Res..

[67] Carattoli A., Zankari E., Garcia-Fernandez A., Voldby Larsen M., Lund O., Villa L., Moller-Aarestrup F., Hasman H. (2014). In silico detection and typing of plasmids using PlasmidFinder and plasmid multilocus sequence typing. Antimicrob. Agents Chemother..

[68] CLSI (2017). Performance Standards for Antimicrobial Susceptibility Testing.

[69] World Health Organization (WHO) Critically Important Antimicrobials for Human Medicine: 6th Revision 2018. Ranking of Medically Important Antimicrobials for Risk Management of Antimicrobial Resistance due to Non-Human Use. https://www.who.int/publications/i/item/9789241515528.

[70] Vazquez-Armenta F.J., Bernal-Mercado A.T., Tapia-Rodriguez M.R., Gonzalez-Aguilar G.A., Lopez-Zavala A.A., Matinez-Tellez M.A., Hernandez-Oñate M.A., Ayala-Zavala J.F. (2018). Quercetin reduces adhesion and inhibits biofilm development by *Listeria monocytogenes* by reducing the amount of extracellular proteins. Food Control.

[71] Avila-Novoa M.G., Navarrete-Sahagún V., González-Gómez J.P., Novoa-Valdovinos C., Guerrero-Medina P.J., García-Frutos R., Martínez-Chávez L., Martínez-Gonzáles N.E., Gutiérrez-Lomelí M. (2021). Conditions of In Vitro Biofilm Formation by Serogroups of *Listeria monocytogenes* Isolated from Hass Avocados Sold at Markets in Mexico. Foods.

